# Outcomes of Human Papilloma Virus Vaccination in a Private Women Health Clinic in Lebanon

**DOI:** 10.1155/2022/7342061

**Published:** 2022-10-12

**Authors:** Muhieddine Seoud, Iman Jaafar, Rayan Ghanem, Christiane Soubhieh, Abdallah Adra, Anwar Nassar, Ali Khalil

**Affiliations:** American University of Beirut Medical Center (AUBMC), Department of Obstetrics and Gynecology, Beirut, Lebanon

## Abstract

**Objectives:**

The study aims to report on the feasibility and associated adverse events of HPV-Vaccination (HPVV) in a private clinic setting in Lebanon and, when available, the results of subsequent cervical cancer screening.

**Methods:**

Opportunistic HPV vaccination is offered at the Women's Health Center of the AUBMC. We retrospectively reviewed the patients' demographic data, the incidence of adverse events, and their cytological screening.

**Results:**

A cohort of healthy women (*n* = 1013) aged 26.2 years (12–54 years) were opportunistically vaccinated with one of two HPV vaccines; 845 (83.4%) received the quadrivalent vaccine (Q4V), and 151 (14.1%) received the bivalent vaccine (B2V). The majority (75.8%) received three doses while 16% received two doses. Out of these women, 26.3% (267) became sexually active postvaccination (NS2), whereas 17% (174) were sexually active prior to vaccination (SA) and the rest 57% (572) reported no sexual activity (NS1). Among the SA group, 26% (46/147) presented with abnormal cytology at time of vaccination. As for the NS2 women, 5% (14/267) had subsequently abnormal screening within 37 (12–103) months following vaccination.

**Conclusions:**

In this observational study, we report the successful introduction of HPVV with negligible adverse events. The incidence of abnormal cervical cytology was low among our patients.

## 1. Introduction

Of all cancers worldwide, 4.5% are attributable to HPV (8.6% in women versus 0.8% in men) [1, 2]. Cervical cancer accounts for 83% of HPV-attributable cancers, two-thirds of which occur in developing countries [1–3]. In Lebanon, a middle-income country, the age-specific incidence rate (ASIR) of cervical cancer is 4.7/per 100,000 [4]. Overall, the Extended Middle East and North Africa (EMENA) region contribute to less than 2% of cervical cancer deaths worldwide, although systematic cancer registration is lacking in many countries of the region [5, 6].

Secondary prevention via cytological testing, despite its low sensitivity, and more recently, HPV DNA testing, with its higher sensitivity, have both contributed to the significant reduction in the incidence and mortality of cervical cancer. However, most countries in the EMENA region, including Lebanon, lack organized screening [6]. The time lag between the peak incidence of HPV infection and the peak incidence of cervical cancer is estimated to be around 2–4 decades, which strongly supports primary preventive strategies via HPV vaccination [7]. The Food and Drug Administration (FDA) in the USA approved the quadrivalent Vaccine (4V): GARDASIL® (Merck), the bivalent Vaccine B2V: CERVARIX™ (GSK), and more recently the nanovalent Vaccine (N9V): GARDASIL® 9 (Merck) as a primary means of preventing HPV-related diseases and cancers [8]. Data continues to support the safety and efficacy of these vaccines, with persistent effectiveness lasting more than 12 years postvaccination [9]. The Center for Disease Control (CDC) Advisory Committee on Immunization Practices (ACIP) recommends that children aged 9–14 years receive only two doses of the HPV vaccine at least six months apart. More recently, the FDA updated its recommendations to include gender-neutral vaccination up to the age of 45 [10, 11]. The efficacy of these vaccinations ranges between 92 and 96% against precancerous lesions of the cervix, vulva, and vagina among women with no prior HPV infection [12–14].

Unlike the rest of the world, the introduction of organized HPVV has been very slow in the EMENA region, except for Abu Dhabi. In Lebanon, data on HPV vaccine uptake is lacking, as well as demographic variables associated with vaccine uptake and the continuation rate for all three doses [15]. In addition, Middle Eastern societies have one of the lowest prevalences of HPV and cervical cancer in the world and embrace a conservative cultural background. Previous data from this part of the world shows that women have a much later age of marriage or sexual activity. Sex activity is limited, with many women presenting to gynecology clinics in Middle Eastern societies who were older yet still not sexually active, hence the older age of vaccination. Systematically organized screening programs are still lacking in Lebanon, including cervical cancer screening [15, 16].

We report on the introduction of HPVV in an adult, private, academic setting in Beirut, Lebanon. We analyze the associated demographic variables, the rate of completion of the three-dose schedule, the incidence of adverse events, and the subsequent incidence of abnormal cytology postvaccination in sexually naïve and sexually active patients.

## 2. Materials and Methods

As of 2007, opportunistic HPVV was offered to women and their adolescent daughters attending the Women's Health Center (WHC) of the American University of Beirut-Medical Center (AUBMC). Following nondirective counseling by physicians and nurse practitioners, women or parents of adolescents chose one of the two available vaccines (Q4V or B2V). The initial purpose of this prospectively collected database was to monitor adverse events (AEs) and to send reminders to vaccine recipients to complete their subsequent doses on time. The patients were followed up as part of the standard of care by the gynecology nurse specialist and called consequently to ensure the completion of HPVV doses. We performed a retrospective review of the electronic medical records of these females from 2007–2017 after obtaining the Institutional Review Board (IRB)'s approval in Beirut (IRB code: OGY.MS.11). Informed consent was waived by the IRB of the American University of Beirut as it did not involve any direct contact with the patients for the aims of the study. The collected data included demographic data such as age, medical history, and residence; the type of vaccine used; batch number; the number of doses received; and any AEs that could have led to the discontinuation of the rest of the vaccination doses. We did not collect data on those who were offered vaccines and refused as per our IRB proposal. In addition, for those women who were or became sexually active, we coupled these data with our cytology laboratory data to report on the rates of subsequent abnormal cytological screening. Initially, all women aged 12 years and <26 years were offered to be vaccinated, but later the age was increased to 45 years.

Women and girls were offered vaccination if they were healthy with no chronic medical illness, not immunocompromised, and not currently treated for cancer, including any genital cancer. A speculum examination was performed in nonvirginal patients and a Pap smear was collected using either an Ayre's spatula or a cytobrush to exclude any lesions suggestive of cancer. Cytological samples were in alcohol and sent to the AUBMC Cytopathology lab, which is accredited by the College of American Pathologists (CAP). 2%) of patients had unsatisfactory cytological samples and were recalled for repeat testing. The cytology slides were read by cytopathologists who were board-certified in Cytopathology by the American Board of Pathology and were unaware of the patients' vaccination status or prior history.

Management of the abnormal screening followed the 2012 ASCCP guidelines [17]. Statistical analysis was performed using SPSS version 25 ( V.25.0). The analysis included Student's *t*-test and chi-square, and statistical significance was considered at *p* < 0.01 and 95% CI.

## 3. Results

From 2007 to 2017, 1013 females were vaccinated with one of the two available HPV vaccines; 845 (83.4%) received Q4V and 151 (14.1%) received B2V. The average age of the recipients was 26.2 ± 5.8 (12–54) years. The majority (75.8%) completed all three doses while only 16% received two doses. Between 2007 and 2012, 88.3% (481/545) completed three doses, while between 2013 and 2017, 74.6% and 25.4% (98/386) received three and two doses, respectively (*p* value <0.001). After 2012, there was a nonsignificant trend toward a two-dose vaccination schedule in both Q4V and B2V (Table 1).  Table 2 reveals the results of the follow-up cytology in NS2 women who were HPV vaccinated with a median follow-up of 37 (12–103) months. Of those who were not initially sexually active, 4.6% (10/219) had subsequent abnormal cytology [ASCUS: 8, LSIL: 2, HSIL/CIS: 0] in the group that received Q4V compared with 8.3% (4/48) [ASCUS: 2, LSIL: 0, HSIL/CIS: 2] in those receiving B2V (*p* = 0.022). The mean age at the first Pap smear in the 267 patients who were or became sexually active was 27.7 ± 4.7 (17–45) years. During that same period, the average rate of abnormal cytology in AUBMC ranged from 1.7 to 2.3% (unpublished institutional data).

**Figure 1:**
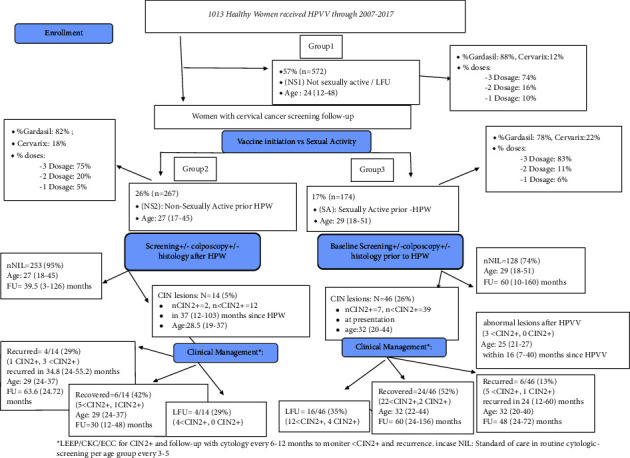
Follow-up of women who received the human papilloma virus vaccine at the Women's Health Center (WHC) of the American University of Beirut-Medical Center (AUBMC) 2007–2017.

Women in the NS2 and SA groups had similar ages at vaccination: [27 (17–45) vs. 29 (18–38), *p* value = 0.06]. Among the 174 women who were SA prior to HPVV (SA), 26% (46) had biopsy-proven CIN; seven were CIN2+ and 39 < CIN2 (Figure 1). Patients were monitored with repeat cytology ± repeat colposcopy at 6- or 12 months posttreatment (HPV DNA testing was not covered by medical insurance companies and was notroutinely done as it was an expensive out-of-pocket test). Management and follow-up were according to the ASCCP 2012 guidelines. Only one of the seven vaccinated patients had a recurrence of the CIN2+ within a two-year follow-up (Figure 1). In the 267 NS2 group, only one of two patients had a recurrence of the CIN2+ within a three-year follow-up. Among patients with treated CIN2+ lesions, the risk of recurrence in the NS2 group was 0.4% (1/267), whereas it was 0.5% in the SA group (1/174) (*p*-value = 0.76, Table 3).

The time to recurrence of all lesions (<CIN2 and CIN2+) in NS2 and SA groups was not statistically significantly different (*p* value = 0.50, Table 4). The time to recurrence of the CIN2+ in the NS2 and SA groups was also not statistically significantly different [34.8 (24–55.2) vs. 24 (12–60) months, respectively, *p*-value = 0.53].

Only one patient had a severe allergic reaction (anaphylactic shock) that included a major reaction that included edema of the face and inability to breath following the administration of the first dose of Q4V; otherwise, there were no other AEs, namely, syncope or reported local injection site reactions.

## 4. Discussion

The prevalence of HPV infection among women in the Middle East and Lebanon is estimated to be around 4–10% [15, 18–21]. A prior study from the same medical center in 2002 reported that, among 1026 healthy women (average age: 40 ± 11.3 years) attending the WHC, 31 (3%) had a high-risk HPV detected during their routine screening [21]. The estimated prevalence of HPV infection in Lebanon and neighboring countries is expected to increase because of the notable change in sexual behavior and practices [22]. Moreover, there is a lack of organized screening programs [15], a false perception among many Lebanese as being at low risk of acquiring a STI, and parental and healthcare providers' hesitance towards HPVV [22–24]. For these reason, among other, most women with cervical cancer are still diagnosed at an advanced stage (unpublished institutional data).

According to the World Health Organization (WHO), the highest impact on the mortality of cervical cancer will most likely occur when HPV vaccination is offered in an organized universal fashion where more than 90% of eligible women are vaccinated [9]. In Australia, organized, universal, and government-supported school-based HPVV was shown to significantly decrease the incidence of genital warts and cervical intraepithelial neoplasia [25]. However, because of the low incidence of cervical cancer in EMENA, the socio-cultural connotation of STI-related vaccination, numerous competing health priorities, and budget constraints, governments have been very reluctant to embark on such national vaccination campaigns [5]. Therefore, it is usually left to individual institutions and individual physicians in the private sector to use opportunistic vaccination to fill this gap.

However, our experience still reflects a successful introduction of HPVV in a private setting in a country with a relatively low incidence of HPV-related diseases. Because it is a Women's Health Center catering to a mostly adult population and not a pediatric clinic setting, the average age of the vaccinated women is much older than in typical organized school-based vaccination campaigns. It is noteworthy that following the release of the data on the efficacy of the two doses of the vaccine in younger girls in 2013, some older girls and women chose to skip the third dose. Nevertheless, the overall uptake of the three-dose regimen remained high. There were no noticeable or reportable AEs except for only one side effect of vaccination found in our study. The interval from the time of vaccination until the performance of the first Pap smear decreased over time, and the subsequent rate of abnormal Pap smears with both vaccines was rather low. This rate, however, is higher than in the overall population of the AUBMC (1.7–2.0%). These findings may be attributed to an earlier onset of sexual activity after vaccination, or maybe women were getting vaccinated because they were intending to get married or become sexually active, or they may reflect an earlier screening pattern over the years. At our institution, the current high cost of HPV screening ($USD 170) is still a major obstacle to using it as a primary screening tool since it is not covered by most third-party payers and most women have to pay the cost of screening out-of-pocket.

It is hard from this small sample and the short follow-up duration of an average of up to 4.5 years, to estimate the impact of vaccination in both the SA and NS2 groups. Thus, a larger sample size is needed to assess the protective effect of the vaccine.

Moreover, the incidence of adverse events was very small except for the anaphylactic reaction following the first dose of the Q4V, which was reported with the batch number at that time to the manufacturer.

The strength of the study is that it is the first comprehensive review of the introduction of HPV vaccination in adult females and adolescent girls in Lebanon and the region with a relatively high continuation rate and low rate of adverse events. The reporting of the follow-up cytological screening, although over a short period, is also noticeable. The limitations of the study are the unknown HPV status of women at baseline and in a country with limited resources and the high cost of HPV testing. Moreover, the study confirms the low rate of CIN2+ lesions in both the AS and the NS2 patients and the low recurrence rates postvaccination as reported [26].

In summary, HPVV can be safely introduced in a private WHC setting, although it does not replace a universal organized vaccination campaign at a younger age. It can be used as an example of a successful catch-up campaign. The high three-dose-schedule continuation rate reflects the high level of education of our patients and the effectiveness of the WHC recall system to ensure the completion of all three doses of the vaccine.

## Figures and Tables

**Table 1:** Distribution of the human papilloma virus vaccination for women and their adolescent girls attending the Women's Health Center (WHC) of the American University of Beirut-Medical Center (AUBMC) 2007–2017.

Year intervals	Doses	Age ≤25	Age >25	Total	*p* value
***2007–2012***	2 doses	26% (15/47)	74% (42/57)	11.6% (57/493)	
	3 doses	42% (184/436)	58% (252/436)	88.4% (436/493)	
Total		40% (199/493)	60% (294/493)	493	

***2013–2017***	2 doses	48% (44/92)	52% (48/92)	25% (92/367)	0.091
	3 doses	58% (160/275)	42% (115/275)	75% (275/367)	
Total		56% (204/367)	44% (163/367)	367	

***2007–2017***	2 doses	40% (59/149)	60% (90/149)	17% (149/860)	0.058
	3 doses	48.4% (344/711)	51.6% (367/711)	83% (711/860)	
	Total	47% (403/860)	53% (457/860)	860	

**Table 2:** Cervical cytology screening in women who were not initially sexually active and received the human papilloma virus vaccine at the Women's Health Center (WHC) of the American University of Beirut-Medical Center (AUBMC) 2007–2017.

		Bivalent vaccine	Quadrivalent vaccine	Total	*p* value
*Age at vaccination (years)*	Mean ± SD	25.9 ± 4.9	28 ± 4.7	27.7 ± 4.7	0.003
	Median (range)	25 (17–38)	27 (18–45)	27 (17–45)	
	*N* (%)	48 (18%)	219 (82%)	267	
*% Of ladies >25 years old at first dose of vaccination*		47.9%	70.3%	66.3%	0.003
		(23/48)	(154/219)	(177/267)	
*Age at first pap smear after vaccination (years)*	Mean	27.7 ± 3.9	29.2 ± 5.0	29.0 ± 4.9	0.057
	Median (range)	28 (18–38)	28 (19–46)	28 (18–46)	
	N	48	219	267	
*% Of ladies >25 years old at first Pap smear*		70.8%	79%	77.5%	0.25
		(34/48)	(173/219)	(207/267)	
Incidence of abnormal Pap smear postvaccination	*N* (%)	(4/48) 8%	(10/267) 4.6%	(14/267) 5%	0.022
*Age at abnormal pap smear postvaccine (years)*	Mean	31.3 ± 3.9	32 ± 4.0	32 ± 3.9	0.637
	Median (range)	32 (26–35)	32 (26–40)	32 (26–40)	
% Of females >25 years old at time of vaccination with subsequent abnormal Pap smear		50% (2/4)	60% (6/10)	57.1% (8/14)	0.733

*Subsequent Pap smear results following HPV vaccination*					
Normal	253	91.7% (44/48)	95.4% (209/219)	94.8% (253/267)	0.022
ASCUS	10	4.2% (2/48)	3.7% (8/219)	3.7% (10/267)	
LSIL	2	0%-(0/48)	1.0% (2/219)	0.75% (2/267)	
HSIL/CIS	2	4.2% (2/48)	0.0% (0/219)	0.75% (2/267)	

**Table 3:** Recurrence of CIN lesions in women who received the human papilloma virus vaccine at the Women's Health Center (WHC) of the American University of Beirut-Medical Center (AUBMC) 2007–2017.

	Outcomes of lesions			*p* Value	Relative risk for recurrence CIN2+
	CIN2+	<CIN2+	Total		
NS2^*∗*^ group (*n* = 267)	1	9	10	0.99	0.3% (1/267)
SA^+^ group (*n* = 174)	1	29	30		0.5% (1/174)

^*∗*^NS2: not previously sexually active at the time of first dose of vaccination; +SA: sexually active before first dose of vaccination.

**Table 4:** Time to recurrence of CIN lesions following treatment of CIN2+ or detection of <CIN2 in women who received the human papilloma virus vaccine at the Women's Health Center (WHC) of the American University of Beirut-Medical Center (AUBMC) 2007–2017.

Recurrence time for CIN2+ and <CIN2+		*p* value
^*∗*^NS2 group (*n* = 4)	37.6 ± 15	0.50
^+^SA group (*n* = 6)	30.1 ± 18	

^*∗*^NS2: not previously sexually active at the time of first dose of vaccination; +SA: sexually active before first dose of vaccination.

## Data Availability

The data are available upon reasonable request from the corresponding author.
